# An efficient and cost-effective method for DNA extraction from athalassohaline soil using a newly formulated cell extraction buffer

**DOI:** 10.1007/s13205-016-0383-0

**Published:** 2016-02-13

**Authors:** Avinash Narayan, Kunal Jain, Amita R. Shah, Datta Madamwar

**Affiliations:** BRD School of Biosciences, Sardar Patel University, Vadtal Road, Satellite Campus, Bakrol, 388 315 Anand, Gujarat India

**Keywords:** Environmental DNA, Saline desert soil, DNA extraction, Athalassohaline, Microbial cell extraction

## Abstract

The present study describes the rapid and efficient indirect lysis method for environmental DNA extraction from athalassohaline soil by newly formulated cell extraction buffer. The available methods are mostly based on direct lysis which leads to DNA shearing and co-extraction of extra cellular DNA that influences the community and functional analysis. Moreover, during extraction of DNA by direct lysis from athalassohaline soil, it was observed that, upon addition of poly ethylene glycol (PEG), isopropanol or absolute ethanol for precipitation of DNA, salt precipitates out and affecting DNA yield significantly. Therefore, indirect lysis method was optimized for extraction of environmental DNA from such soil containing high salts and low microbial biomass (CFU 4.3 × 10^4^ per gram soil) using newly formulated cell extraction buffer in combination with low and high speed centrifugation. The cell extraction buffer composition and its concentration were optimized and PEG 8000 (1 %; w/v) and 1 M NaCl gave maximum cell mass for DNA extraction. The cell extraction efficiency was assessed with acridine orange staining of soil samples before and after cell extraction. The efficiency, reproducibility and purity of extracted DNA by newly developed procedure were compared with previously recognized methods and kits having different protocols including indirect lysis. The extracted environmental DNA showed better yield (5.6 ± 0.7 μg g^−1^) along with high purity ratios. The purity of DNA was validated by assessing its usability in various molecular techniques like restriction enzyme digestion, amplification of 16S rRNA gene using PCR and UV–Visible spectroscopy analysis.

**Electronic supplementary material:**

The online version of this article (doi:10.1007/s13205-016-0383-0) contains supplementary material, which is available to authorized users.

## Introduction

The molecular analysis of community DNA is the ultimate route to study the diversity of microbial wealth and genetic variation in natural conditions, to recover novel genes for understanding their metabolic functions, to track metabolic pathways and genetic adaptations for surviving under various environmental conditions (Kakirde et al. 2010; Delmont et al. 2012; Qu et al. 2009; Cary et al. 2010; Sharma et al. 2014). Subsequently, extraction of highly pure and unbiased environmental DNA is very fundamental and significant process. It requires basic understanding of physicochemical properties of soil (viz. organic content, presence of metal ions, salts, etc.) that always hinders the effectiveness of various treatment procedures and chemicals used during DNA extraction, which inturn affects the quality and quantity of extracted environmental DNA (Lombard et al. 2011; Young et al. 2014). Moreover, every environmental sample has its own set of physicochemical composition and biomass abundance. Therefore, every type of soil needs protocol optimization for environmental DNA extraction.

Many studies have demonstrated the extraction of environmental DNA from different types of environments. Various approaches like direct lysis, freeze–thaw lysis (Herrick et al. 1993), bead beating (Miller et al. 1999; Courtois et al. 2001; Petric et al. 2011; Urakawa et al. 2010), liquid nitrogen grinding (Ranjard et al. 1998), ultrasonication (Picard et al. 1992), hot detergent treatment (Holben 1994), use of strong chaotropic agents like guanidinium salts (Porteous et al. 1997), and high concentration of lysozyme treatment (Hilger and Myrold 1991) have been applied for environmental DNA extraction. Generally, the direct lysis method is believed to cause DNA shearing and also fails to remove impurities including humic acid, fulvic acid, metal ions and salts, the major interfering agents in molecular analysis. Therefore, it needs additional purification step, which ultimately lowers the DNA yield. Moreover, the biasness and shearing effect due to direct lysis method limit the use of environmental DNA in large insert-based library preparation and also its use on the next generation sequencing platform. Therefore, developing an indirect lysis method is the utmost requirement for metagenomics-mediated community analysis. Many reports clearly describe the advantages of indirect methods over direct lysis method (Zapata et al. 2010; Qiao et al. 2013; Delmont et al. 2011). Since microbial cell extraction is the key step of the indirect lysis method, the formulation of extraction buffer and the primary need to establish indirect lysis-based DNA extraction protocol are highly imperative.

In the present study, we have demonstrated an indirect lysis-based DNA extraction method by formulating PEG-NaCl-based cell extraction buffer with a promising efficiency of microbial cell extraction/recovery from athalassohaline soil samples. PEG is amphiphilic in nature, which thought to interact with cells and it was observed that cell wall absorbs high amounts of PEG that may be responsible for cell dissociation from soil particles. The success of the protocol was verified by comparing the quality and quantity of extracted environmental DNA with that of three commercially available DNA extraction kits namely NucleoSpin Soil, ZR soil Microbe DNA (both kits are based on direct lysis) and XcelGen Soil gDNA Isolation kit (based on indirect lysis), and three widely used DNA extraction methods, beat beating method (Miller et al. 1999), hot detergent lysis method (Desai and Madamwar 2006) and indirect lysis method employing high concentration of lysozyme with hot detergent lysis (Gabor et al. 2003).

## Materials and methods

### Soil sampling

Subsurface (8–10 cm) soil core samples were collected from four distinct sites from Great Rann of Kachchh, Gujarat, India, designated as: BOP-Dharamshala (24°2′4″N, 69°39′46″E), India Bridge (23°59′13″N, 69°44′41″E); Near India Bridge (23°59′35″N, 69°42′12″E) and Vighakot (24°13′1″N, 69°11′51″E). Soils were sieved (~2 mm poresize) for removing coarse particles and other debris and plant roots. The sieved soil samples were stored at 4 °C under dark conditions. Soil characteristics were determined through standard methods.

### Buffers

Cell extraction buffer: 1 % (w/v) PEG 8000, 1 M NaCl, pH of the buffer was adjusted to pH 9.2, using 0.2 N NaOH.

Suspension buffer: 10 % Sucrose, 10 mM Tris–Cl (pH 8.0), 50 mM EDTA (pH 8.0), 50 nM NaCl.

TE buffer: 10 mM Tris (pH 8.0); 1 mM EDTA (pH 8.0).

### Extraction of microbial cells

Five hundred milligrams of soil from each site were suspended in 50 ml of newly formulated cell extraction buffer. The soil suspension was continuously mixed for 3 min at 25 °C on tube rotator (SLM-TR-100, Bangalore GeNei) with the speed of 16 rpm. This homogenous mixture was centrifuged at lower speed of 220×*g* for 5 min at 25 °C. The first centrifugation step at lower speed is essential to retain cell mass in supernatant and to pellet other soil particles to prevent them for co-extraction with cell pellet. The cell mass was harvested at comparatively higher speed of 6500×*g* for 20 min at 25 °C. The obtained cell mass was resuspended in 500 µl of sterile suspension buffer.

### Acridine orange staining for cell extraction efficiency determination

The efficiency of cell extraction was determined by acridine orange staining (0.1 %; w/v, filter sterilized). Each soil samples before and after cell extraction was visualized under an epifluorescence microscope (BX41, Olympus) and cell count for both the sample was measured by manual counting of the fluorescence dots.

### Cell lysis, DNA extraction and purification

DNA was extracted by two-step cell lysis by a combination of chemical, (enzymatic lysis and hot detergent lysis) and physical (bead beating) methods. Initially cell mass was lysed by adding 50 µl of freshly prepared lysozyme (20 mg ml^−1^) and incubated at 37 °C for 45 min under shaking conditions followed by Proteinase K treatment (12.5 µl, 20 mg ml^−1^) at 55 °C for 45 min. The resultant cell lysate was further lysed by SDS treatment (50 µl, 20 %; w/v) at 65 °C for 45 min with intermittent mixing at every 5 min interval. The cell lysate was centrifuged at 11,000×*g* for 3 min at 20 °C; supernatant (S1) was collected and the pellet was resuspended in suspension buffer (200 µl) alongwith 20 % SDS (50 µl) and ~500 mg sterile glass beads (1–1.5 mm) and vortexed at maximum speed for 3 min. The lysate was again centrifuged at 11,000×*g* for 3 min at 20 °C to pellet down cell debris and supernatant (S2) was mixed with S1 and subjected for RNase A (10 µl of 10 mg ml^−1^, 37 °C, 15 min) treatment. Cellular proteins and other cell debris were precipitated through 0.35th volume 2.5 M potassium acetate (pH 8.0). The precipitate was removed by combination of two-step centrifugation of low (6500×*g*, 20 °C, 3 min) and high (8000×*g*, 20 °C, 3 min) speed. Metagenomic DNA was precipitated from the aqueous phase by adding equal volume of isopropanol and incubated for 5 min under ambient conditions and DNA precipitate was pelleted at 11,000×*g*, at 4 °C for 20 min. DNA pellet was washed twice with freshly prepared 70 % ethanol, dried at 55 °C for 10 min and resuspended in 50 µl nuclease free TE buffer and stored at −20 °C till further use.

### DNA quantification, purity and spectroscopic analysis

Extracted DNA was quantified on Nanodorp spectrophotometer (Implen GmbH, Germany) and its purity was expressed as ratios of absorption at A_260_/A_280_ and A_260_/A_230_. Moreover, the diluted (1:10 in TE buffer) DNA samples analyzed over 230–260 nm using UV–Visible spectrophotometer (Specord 210, Analytik Jena AG, Jena, Germany).

### 16S rRNA gene amplification

The above DNA extraction method was validated by accessing its purity and amenability for further molecular analysis by amplifying 16S rRNA gene through polymerase chain reaction (PCR). The extracted DNA was used as a template (~50 ng) in a 30 µl reaction system containing 1 X reaction buffer (10 mM Tris–Cl, pH 9.0, 15 mM MgCl_2_, 0.1 % Triton X-100), 0.30 mM of each dNTPs, 0.60 pmol of each universal primers 8F (5′-AGA GTT TGA TCC TGG CTC AG-3′) and 1492R (5′-GGT TAC CTT GTT ACG ACT-3′) and 1.5 units of Taq DNA polymerase. 16S rRNA gene was amplified through initial denaturation at 94 °C for 4 min, followed by 30 cycles of denaturation at 94 °C for 1 min, primers annealing at 54 °C for 1 min and extension at 72 °C for 1 min and final extension at 72 °C for 5 min. Gene amplification was observed by electrophoresis of amplified products on 1.2 % agarose in 1 X TAE buffer [40 mM Tris acetate, 1 mM EDTA (pH 8.0)].

### Comparison of DNA extraction method

The efficiency of newly developed environmental DNA extraction method was compared with extraction from same soil samples with three commercially available kits (a) NucleoSpin^®^ Soil (Macherely-Nagel GmbH, Germany), (b) ZR Soil Microbe DNA MiniPrep (Zymo Research, USA) and (c) XcelGen Soil gDNA isolation (based on indirect lysis) (Xcelris Genomics, India) and three widely used manual protocols (d) hot detergent lysis and column purification (Desai and Madamwar 2006), (e) bead beating lysis (Miller et al. 1999) and (f) high concentration of lysozyme/hot detergent lysis (Indirect lysis method) (Gabor et al. 2003). Environmental DNA extracted by above methods was compared with that of newly developed method in terms of purity, yield and quality by using UV–Visible spectroscopy, restriction enzyme digestion, and polymerase chain reaction amenability.

## Results and discussion

### Soil characteristics

Results from Table S1 revealed the saline nature of the soils of Rann of Kachchh with average electrical conductivity of 2.02 μS cm^−1^ and measured salinity at the level of 8.85 ppt. Soil evidently contains comparatively high amount of metal ions and salts viz, calcium 262 mg kg^−1^, magnesium 126.6 mg kg^−1^, sodium 163 mg kg^−1^, chloride 311 mg kg^−1^, etc. It is understood that metal ions and salts have a tendency to bind DNA and cell surface receptors, thereby preventing direct DNA extraction from such soils and it co-precipitates along with DNA as DNA-salt complex, which in turn inhibits down stream DNA processing. However, Eichhorn and Shin (1968) observed that the negatively charged DNA strands tend to unwind in the absence of counter ions.

### Microbial cell extraction and extraction efficiency

Since the present DNA extraction method was primarily based on cell extraction, the composition of cell extraction buffer plays an important role for obtaining better DNA yield. Cell extraction from saline soil by newly formulated and optimized cell extraction buffer showed better DNA yield and maximum DNA purity along with combination of low and high speed centrifugation. PEG 8000 helps in dissociation of cells from soil particles, whereas NaCl increases the cell stability by preventing osmotic lysis. Moreover, at low speed centrifugation (220×*g*) coarse soil particles were removed and at high speed centrifugation (6500×*g*) cell mass was harvested for DNA extraction.

**Fig. 1:**
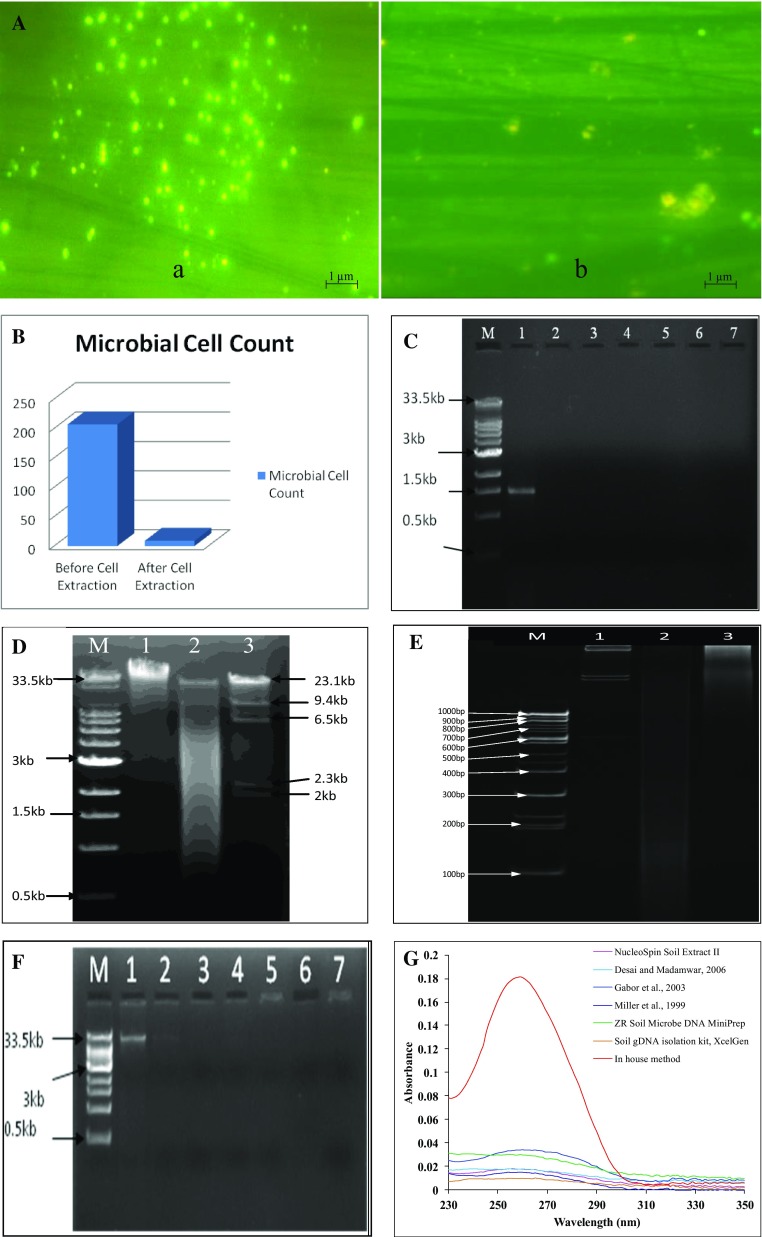
**A** Epifluorescence microscopic images of acridine orange stained slides, (*a*) intact soil sample and (*b*) soil sample after cell extraction. **B** Acridine orange staining-based microbial cell count by epifluoresence microscopy before and after cell extraction. **C** Electrophoresed 1 % gel showing amplified 16S rRNA gene, *M* molecular weight marker, *lane 1*: amplified 16S rRNA gene from environmental DNA extracted by newly developed method, *lane 2–7*: amplification of 16S rRNA gene from DNA extracted by published methods and kits, (it can be observed that DNA was unable to amplify by these methods). **D** Electrophoresed 1 % agarose gel showing, *lane M*: SuperMix DNA ladder (0.5 kb to 33 kb), *lane 1*: environmental DNA, *lane 2*: mixture of environmental DNA extracted from newly developed method and *λ* DNA digested with *Hind* III, *lane 3*: λ DNA digested with *Hind* III and. **E** Polyacrylamide gel (9 %) showing: *lane M* molecular weight marker 100 bp, *lane 2*: λ DNA digested using *Hind* III, *lane 3*: completely digested environmental DNA extracted with newly developed method with *Hae* III (10 h), and *lane 4*: partially digested environmental DNA extracted with newly developed method with *Hae* III (1 h). **F** Electrophoresed 1 % agarose gel showing DNA marker and extracted environmental DNA by various methods. *M* denotes molecular weight marker, *lane 1*: showing environmental DNA extracted by newly developed method, *lane 2*: high concentration of lysozyme lysis method (Gabor et al. 2003), *lane 3*: hot detergent lysis method (Desai and Madamwar 2006), *lane 4*: bead beating *lane* (Miller et al. 1999) *5*: NucleoSpin Soil Extract II, *lane 6*: Soil gDNA isolation kit (XcelGen), *lane 7*: ZR Soil Microbe DNA MiniPrep. **G** UV–visible absorbance spectra of environmental DNA extracted by described mentioned methods and kits

Figure 1A demonstrates the photographic images of acridine orange staining of the soil before and after cell extraction and Fig. 1B shows comparative account of cell count before and after treatment. The direct count of cell in intact soil and cell extracted soil by epifluorescence microscopy showed that the cell extraction efficiency of the extraction buffer is nearly 95 %. The observed results suggested that the cell extraction efficiency was relatively better and higher, on comparing with nycodenz based microbial cell extraction method, extracting only 50 % of the cells (Robe et al. 2002).

### DNA quantification and purity

It was observed that during DNA extraction from saline soils, co-extracted salts and other complex compounds like humic acids are major impurities which not only decrease the total DNA yield but also prevent other in vitro molecular reactions. Results from the Table 1 showed that newly developed indirect DNA extraction method yielded 5.6 ± 0.7 µg of metagenomic DNA per gram of saline soil with purity ratios of 1.820 for A_260_/A_280_ and 1.732 for A_260_/A_230_.

**Table 1:** Comparison of purity ratio, DNA yield and PCR amenability of environmental DNA extracted by newly developed method and other recognized methods and commercial kits

Method	A_260/280_	A_260/230_	Average DNA yield (µg g^−1^)	PCR amenability
Indirect lysis (newly developed method)	1.820	1.732	4.6	+
Indirect lysis (Gabor et al. 2003)	1.512	0.952	1.0	−
Direct lysis (Desai and Madamwar 2006)	–	–	–	−
Direct lysis (Miller et al. 1999)	–	–	–	−
Direct lysis (NucleoSpin^®^ soil)	1.657	0.714	0.3	−
Direct lysis based (ZR soil microbe DNA MiniPrep)	1.500	0.432	0.5	−
Indirect lysis based (XcelGen soil gDNA isolation)	1.677	0.815	0.2	−

Metagenomic DNA extracted by the method developed by Gabor et al. (2003) also gave good purity ratios; however, DNA yield was very low (Table 1). It was observed that the spectrophotometric measurements for DNA quality assessment with higher values associated with better DNA purity (Psifidi et al. 2015).

The purity level of the extracted DNA was accessed by amplifying 16S rRNA using extracted DNA as template and restriction digestion by *Hind* III. Figure 1C demonstrates the amplified products of ~1.5 kb of 16S rRNA gene from extracted DNA using newly developed method, while Fig. 1D, E, shows the catalytic breakdown of metagenomic DNA by restriction enzyme *Hind* III on 1 % agarose and 9 % polyacrylamide gel, respectively. Environmental DNA, extracted by present methods also gave good results when analyzed on the Illumina MiSeq Platform for microbial community structure analysis. Thus, the above results evidently suggested that the efficiency, productivity and level of purity of DNA extracted by newly developed method are significantly higher and it can be used for routine DNA extraction from saline soils.

### Comparison of extraction method

It was observed from Fig. 1F that DNA extracted from three commercial kits and two protocols developed previously (Miller et al. 1999; Desai and Madamwar 2006) was unable to extract any detectable amount of environmental DNA from soils of Rann of Kachchh. However, very low yield of metagenomic DNA was obtained, but with higher purity ratios (as mentioned in “DNA quantification and purity”) by indirect lysis method developed by Gabor et al. (2003). Figure 1G demonstrates the overlay graph of absorbance between 230 and 350 nm for the DNA extracted by all six methods. The results clearly revealed the better productivity and efficiency of newly developed protocol over other established methods and commercially available kits.

## Conclusion

The presented protocol was highly efficient for metagenomics DNA extraction athalasohaline soil. To the best of our knowledge, the study first time demonstrated the use of PEG 8000 in combination of 1 M NaCl at pH 9.2 for the extraction of microbial cell biomass from the soil. The purified environmental DNA was highly compatible for further molecular analysis like PCR amplification, restriction enzyme digestion and community analysis by next generation sequencing technology.

## Electronic supplementary material

Below is the link to the electronic supplementary material.

Supplementary material 1 (DOCX 12 kb)
