# Supplementary material for: An efficient and cost-effective method for DNA extraction from athalassohaline soil using a newly formulated cell extraction buffer
Source: 3 Biotech. 2016 Feb 13;6(1):62. doi: 10.1007/s13205-016-0383-0 (PMC4752949; doi:10.1007/s13205-016-0383-0)
Supplement: Supplementary file 1 — Supplementary material 1 (DOCX 12 kb) [file 13205_2016_383_MOESM1_ESM.docx]

| **Parameters** | **Unit** | **Soil sample of Saline Desert** |
| --- | --- | --- |
| **Physical** |  |  |
| pH | - | 7.22 |
| Electrical conductivity | µS cm^-1^ | 2.02 |
| Moisture | % | 2.09 |
| Bulk density | - | 1.41 |
| Specific gravity | - | 0.95 |
| **Chemical** | | |
| Calcium | mg kg^-1^ | 262.58 |
| Cation Exchange capacity | Meq L^-1^ | 18.45 |
| Chloride | mg kg^-1^ | 311.51 |
| Lithium | mg kg^-1^ | 45.66 |
| Magnesium | mg kg^-1^ | 126.6 |
| Phosphorus | mg kg^-1^ | 0.04 |
| Potassium | mg kg^-1^ | 28.07 |
| Salinity | ppt | 8.85 |
| Sodium | mg kg^-1^ | 163.88 |
| Sulphate | mg kg^-1^ | 53.72 |
| Total Organic Carbon | % | 0.81 |
| Copper | mg kg^-1^ | 23.92 |
| Iron | mg kg^-1^ | 223.41 |
| Zinc | mg kg^-1^ | 3.41 |

**Supplementary Table 1:** Physico-chemical characteristics of soil samples collected from Rann of Kachchh
